# Production, purification and characterization of an acid/alkali and thermo tolerant cellulase from *Schizophyllum commune* NAIMCC-F-03379 and its application in hydrolysis of lignocellulosic wastes

**DOI:** 10.1186/s13568-018-0696-y

**Published:** 2018-10-17

**Authors:** Bikash Kumar, Nisha Bhardwaj, Ansar Alam, Komal Agrawal, Himanshu Prasad, Pradeep Verma

**Affiliations:** 1 Bioprocess and Bioenergy Laboratory, Department of Microbiology, Central University of Rajasthan, NH-8, Bandarsindari, Kishangarh, Ajmer, Rajasthan 305817 India; 2 Department of Life Sciences & Bioinformatics, Assam University, Silchar, Assam 788011 India

**Keywords:** *Schizophyllum commune*, Cellulase, Response surface methodology, Aqueous two-phase system, Hydrolysis

## Abstract

A cellulase producing fungus *Schizophyllum commune* NAIMCC-F-03379 was isolated from decomposed leaf sample of *Lantana camera.* The nutritional components (wheat bran, magnesium sulphate and calcium chloride concentrations) and physical parameters (temperature and pH) were optimised by response surface methodology for enhanced cellulase production by *S. commune* NAIMCC-F-03379. The optimized medium contained: 1% (w/v) wheat bran, 0.3 g/L MgSO_4,_ 0.8–1.0 g/L CaCl_2_, optimum temperature and pH were 25 °C and 5 respectively. Under optimum condition, 5.35-fold increase in CMCase and 6.62-fold increase in FPase activity was obtained as compared to un-optimized condition. Crude cellulase enzyme was subjected to different purification techniques and comparative evaluation of their efficiency was performed. The aqueous two-phase system using polyethylene glycol 8000/MnSO_4_ system showed maximum purification with 10.4-fold increase in activity, 79.5% yield and 0.5 partition coefficient. The cellulase enzyme obtained from *S. commune* NAIMCC-F-03379 has shown high stability i.e. more than 55% relative activity after 12 h of incubation over wide range of temperature (25–65 °C) and pH (3–10). The molecular weight of the cellulase enzyme was estimated as ~ 60 kDa by using sodium dodecyl sulphate-polyacrylamide electrophoresis (SDS-PAGE) and zymography. K_m_ and V_max_ value of cellulase on carboxy-methyl cellulose were obtained as 0.0909 mg/mL and 45.45 μmol/min mg respectively. Rice straw and wheat bran were subjected to hydrolysis using cellulase and cellulase–xylanase cocktail and analysed by thin layer chromatography and high performance liquid chromatography (HPLC). The HPLC analysis showed glucose concentration of 1.162 mg/mL after enzymatic hydrolysis of rice straw.

**Electronic supplementary material:**

The online version of this article (10.1186/s13568-018-0696-y) contains supplementary material, which is available to authorized users.

## Introduction

The environmental pollution caused by use of fossil fuels and constantly increasing energy crisis has stimulated the researchers for developing methods to utilize renewable resources for energy generation. Lignocellulosic biomass (LB) has emerged as a promising alternative to fossil fuels (Pang et al. 2010). It consists of lignin, cellulose and hemicellulose, among which cellulose and hemicellulose represents most abundant, un-digestible by human and unexploited renewable resources (Coman and Bahrim 2011; Kumari and Singh 2018). Cellulose is a water insoluble polysaccharide which is made up of glucose monomers linked by β-1,4 glycosidic bonds. These glucose monomers can be efficiently converted into bioethanol and several value-added chemicals  via  the commercially available chemical processes or fermentation using microbes (Chaturvedi and Verma 2013). The conversion of cellulosic bio-waste to food, feed and energy resources is hindered by its complex microcrystalline structure (Premalatha et al. 2015). In order to overcome these structural complexities and achieve efficient hydrolysis of cellulose various conventional strategies were used such as  treatment by mineral acids, ionic liquid promoted dissolution, sub- or super-critical water. Those conventional techniques are costly, results in the corrosion of vessels apart from problems associated with waste generated during the process (Pang et al. 2010). Therefore, to overcome the limitations and minimize the environmental impact, in recent times a green approach of utilizing cellulase enzyme complex is suggested. A group of glycosyl hydrolases i.e. endoglucanase (endo-1,4-β-d-glucanase EG, EC 3.2.1.4) which act internally on the chain of cellulose cleaving linked bond liberating non- reducing ends, cellobiohydrolase (exo-1,4-β-d-glucanase, CBH, EC 3.2.1.91) that remove cellobiose from this non-reducing end of cellulose chain (Li et al. 2012) and β-glucosidase (1,4-β-d-glucosidase, BG, EC 3.2.1.21) completes the hydrolysis by splitting cellobiose and small cello-oligosaccharides to glucose and are collectively called as cellulase enzyme complex. The cellulase enzyme complex causes selective breakdown of cellulosic biomass to its constituent’s monomeric units which can acts as raw materials for various industries such as food, chemicals (Gomaa 2013) animal feed (Premalatha et al. 2015), paper and pulp industry and bioethanol (Moreau et al. 2015; Srivastava et al. 2018).

The cellulase enzymes are produced naturally by various type of microbes such as fungi (Sohail et al. 2009; Ellilä et al. 2017), bacteria (Saratale et al. 2010; Nargotra et al. 2016; Grigorevski de Lima et al. 2005). Fungus *Trichoderma* spp. and bacteria *Bacillus* spp. are the most intensively studied and used for cellulase production on commercial scale (Kuhad et al. 2011).

The cost of substrate and low enzyme yield are main obstacles in the economics of the cellulase production, which negatively affect its potential for their application on large scale production. Therefore in order to improve the economics of the cellulase enzymes production, agro-residues such as wheat bran (~ 30% cellulosic content) can act as inexpensive alternative to costly substrate such as cellulose or avicel (Knob et al. 2014). Similarly, the limitations associated with low enzyme yield can be overcome by either introducing more suitable microbial species or inducing cellulase production by optimization of parameters controlling the enzyme yields (Singh et al. 2014a). The extracellular enzyme production can be controlled by nutritional composition of media such as mineral salts, carbon source (Zambare 2011; Aanchal et al. 2016) and physical process parameters such as incubation period, temperature and pH (Goyal et al. 2014; Yang et al. 2014) and these factors play significant role in development of industrial bioprocess for enzyme production (Sadhu et al. 2014; Premalatha et al. 2015; Hu et al. 2018). The RSM is a statistical experimental design which involves factorial design and regression analysis that helps in evaluating the efficient parameters along with their interactions in order to find the optimum conditions of different variables for enhanced enzyme production (Bhardwaj et al. 2017; Irfan et al. 2017; Parkhey et al. 2017).

Thus, the present study involves isolation of fungal strain *Schizophyllum commune* NAIMCC-F-03379 from decomposed leaf sample from forests of Cachar district Assam. Statistical optimization of physical and nutritional parameters for enhanced production of cellulase from *S. commune* NAIMCC-F-03379 using RSM was performed. The crude enzyme was purified using different strategies and a comparative evaluation of purification efficiency of each method was performed. The cellulase was subjected to characterization followed by SDS-PAGE and zymography. The potential of cellulase for the hydrolysis of agro-residues was evaluated by TLC and further confirmed by HPLC.

## Materials and methods

### Isolation, screening and identification of cellulase producing fungal strain

The fungal strain was isolated from decomposed leaf sample of *Lantana camera* collected from forest in Cachar district of Assam, India. They were maintained on potato dextrose agar (PDA) plates and stored in refrigerator at 4 °C. The isolates were screened for industrially important cellulase enzymes. The screening was done on czapek dox agar plate which was incorporated with 1% of CMC as a carbon source. The inoculated plates were then incubated at 28 °C for 4–6 days based on the growth rate. The 4–6 days incubated fungal plates were flooded with 0.1% congo red solution for 30 min. Plates were washed with distilled water and destained with 1 M sodium chloride solution for 15 min. The plates were then observed and zones of clearance were measured (Meddeb-Mouelhi et al. 2014).

The genomic DNA of the selected strain was extracted by using the protocol of Prabha et al. (2013). PCR amplification and DNA sequencing were performed on the partial rRNA region, as described by Schoch et al. (2012). The partial 18S rRNA SSU-ITS1-5.8SrRNA-ITS2-partial LSU rRNA region was amplified with the primers ITS4 (5′-TCCTCCGCTTATTGATATGC-3′) and ITS5 (5′-GGAAGTAAAAGTCGTAACAAGG-3′). The PCR product was sequenced at Genomics Corp, Xceleris labs (Ahmedabad, Gujarat, India). The strain was identified with ITS sequence analysis using BLASTn search tools (http://www.ncbi.nlm.nih.gov). The phylogenetic tree was constructed by using MEGA-5 software. The sequence for the *S. commune* was submitted to GenBank with accession number MG923341.1. The fasta format of the partial Sequence of the *S. commune* strain internal transcribed region is given follow:

>MG923341.1 *S. commune* strain: GACTGCGGAGACATTAACGAATCAACA AGTTCATCTTGTTCTGATCCTGTGCACCTTATGTAGTCCCAAAGCCTTCACGGGCGGCGGTTGACTACGTCTACCTCACACCTTAAAGTATGTTTACGAATGTAATCATGGTCTTGACAGACCCTAAAAAGTTAATACAACTTTCGACAACGGATCTTTTGGGCTCTCGCATCGATGAAGAACGCAGCGAAATGCGATAAGTAATGTGAATTGCAGAATTCAGTGAATCATCGAATCTTTGAACGCACCTTGCGCCCTTTGGTATTCCGAGGGGCATGCCTGTTTGAGTGTCATTAAATACCATCAACCCTCTTTTGACTTCGGTCTCGAGAGTGGCTTGAAGTGGAGGTCTGCTGGAGCCTAACGGAGCCGCTCCTCTTAAATGTATTAGCGGATTTCCCTTGCGGGATCGCGTCTCCATGTGATAATTTCTACGTCGTTGACCATCTCGGGGCTGACCTAGTCAGTTTCAATAGGAGTCTGCTTCCAACCGTCTCTTGACCGAGACTAGCGACTTGTGCGCTAACTTTTGACTTGACCTCAAATCAGGTAGGACTACCCGCTGAACTTAAG.

### Medium selection for the cellulase enzyme production

Medium selection is very important step in bioprocess. Four different type of liquid medium, czapek dox broth (M1), basal salt medium with wheat bran (M2), CMC synthetic liquid media (M3) and modified Mendel’s and Sternberg’s basal salt (MSBS) medium (M4) were tested for selection of medium. The details of media components are described below:

M1: Czapek dox broth (g/L): sucrose—30.0, NaNO_3_/KNO_3_—2.0, MgSO_4_·7H_2_O—0.5, KCl-0.5, KH_2_PO_4_—1.0, and FeSO_4_·7H_2_O—0.01, pH—5 (Tallapragada and Venkatesh 2011).

M2: Basal salt media with wheat bran (g/L): wheat bran—10.0, yeast extract—5, Na_2_HPO_4_·2H_2_O—2.0, KCl—0.5, FeSO_4_·7H_2_O—0.15, pH—7 (Bakri et al. 2010).

M3: CMC synthetic liquid medium (g/L): CMC—10.0, yeast extract—0.1, (NH_4_)_2_SO_4_—0.5, KH_2_PO_4_—10.0, MgSO_4_·7H_2_O—0.1, KCl—0.2, pH—5 (El-Hadi et al. 2014).

M4: Modified MSBS medium (g/L): CMC—10.0, (NH_4_)_2_SO_4_—3.5, KH_2_PO_4_—2.0, urea—0.3, CaCl_2_—0.3, peptone—1.0, MgSO_4_·7H_2_O—0.3; trace elements (mg/L): FeSO_4_·7H_2_O—5, MnSO_4_·H_2_O—1.6, ZnSO_4_·7H_2_O—1.4, CoCl_2_—1.7 and Tween 80—0.1% (v/v), pH—5, (Shah and Madamwar 2005).

The submerged fermentation was carried out in 250 mL of Erlenmeyer flask containing 100 mL of liquid medium. Medium was inoculated with fungal spore suspension (4.20 × 10^7^ spore/mL) and incubated at 28 °C. Samples were periodically withdrawn at regular intervals of 24 h. The culture broth was centrifuged at 10,000 rpm for 10 min at room temperature, and the supernatant obtained was used for the enzyme assay. The un-inoculated sample was used as a control in each experiment.

### Quantitative assay of the cellulase activity

The CMCase activity and FPase activity measurements were carried out as per method described by Mandels and Weber (1969) using 1% (w/v) CMC and 50 mg whatman filter paper as substrate respectively. One International unit (IU) of enzyme activity is defined as the amount of enzyme which releases 1 micromole of reducing sugar per minute.

### Enzyme production by submerged fermentation using different carbon sources

The isolated fungus was subjected to submerged fermentation. Spore suspension (4.20 × 10^7^ spore/mL) was taken from the 7 day old culture of *S. commune* NAIMCC-F-03379 and inoculated in a 250 mL erlenmeyer flask containing 100 mL of modified MSBS medium M4. The selection of agro-wastes for cellulase production was performed by replacing 1% CMC with 1% of any of agro-waste i.e. rice straw (RS), rice husk (RH), wheat straw (WS), wheat bran (WB), and sugarcane bagasse (SB). The inoculated medium was kept under static conditions at 28 ± 1 °C. Samples were periodically drawn after intervals of 24 h. The culture broth was centrifuged at 10,000 rpm for 10 min at room temperature, and the supernatant obtained was used for the enzyme assay. The un-inoculated sample was used as a control in each experiment.

### Statistical optimization of nutrient and physical parameters for enhanced cellulase production

The statistical software package “Design Expert” for windows version 9.0 (Stat-Ease Inc., Minneapolis, Minnesota, USA) was used for the statistical processing and tabulating procedures, which enable very fast and simple data appraisal (Bhardwaj et al. 2017). This design suggested least number of variable permutations. An advantage of this design is that combinations of variables are appraised at multiple level of another factor. Therefore, valid wrapping up over an ample choice of experimental conditions can be drawn. The optimization of combined interaction of variable nutrient component and physical parameters were optimized by response surface methodology (RSM) using central composite design (CCD). The data obtained from CCD on cellulase production was focused to analysis of variance (ANOVA). CCD results were analysed and the regression model was proposed. Accordingly, the CCD matrixes of 26 experiments (= 2^k−1^ + 2 * k + 1), where k represents the number of variables to be investigated coating full design of five variables with 3 different levels (− 1, 0 and + 1) used for building quadratic models (Table 1).

**Table 1:** Experimental range and levels of the independent variables for the CCD based RSM experiment

Variable	Components	Range	Levels of variable studied		
			Low (− 1)	Centre point (0)	High (+ 1)
A	Wheat bran	0.5–1.5	0.5	1	1.5
B	Magnesium sulphate	0.3–0.7	0.3	0.5	0.7
C	Calcium chloride	0.5–1.0	0.5	0.7	1
D	Temperature	25–45	25	35	45
E	pH	3.0–7.0	3	5	7

### Statistical analysis and modelling

The data obtained from CCD-RSM on cellulase production were subjected to analysis of variance (ANOVA). Results obtained from experiments were used to fit a second-order polynomial Eqs. (1, 2), as it represents the behaviour of such a system more appropriately

1 $$\begin{aligned} {\text{Y}} &=\upbeta_{0} + \upbeta_{ 1} {\text{A}} + - \upbeta_{ 2} {\text{B}} + \upbeta_{ 2} {\text{C}} + - \upbeta_{ 4} {\text{D}} \hfill \\ & \quad + \upbeta_{ 5} {\text{E}} + \upbeta_{ 1} \upbeta_{ 2} {\text{AB}} + \upbeta_{ 1} \upbeta_{ 3} {\text{AC}} + - \upbeta_{ 1} \upbeta_{ 4} {\text{AD}} \hfill \\ & \quad + \upbeta_{ 1} \upbeta_{ 5} {\text{AE}} + \upbeta_{ 2} \upbeta_{ 3} {\text{BC}} + - \upbeta_{ 2} \upbeta_{ 4} {\text{BD}} + \upbeta_{ 2} \upbeta_{ 5} {\text{BE}} \hfill \\ & \quad + - \upbeta_{ 3} \upbeta_{ 4} {\text{CD}} + \upbeta_{ 3} \upbeta_{ 5} {\text{CE}} + - \upbeta_{ 4} \upbeta_{ 5} {\text{DE}} + - \upbeta_{ 1}^{ 2} {\text{A}}^{ 2} \hfill \\ & \quad + - \upbeta_{ 2}^{ 2} {\text{B}}^{ 2} + \upbeta_{ 3}^{ 2} {\text{C}}^{ 2} + - \upbeta_{ 4}^{ 2} {\text{D}}^{ 2} + \upbeta_{ 5}^{ 2} {\text{E}}^{ 2} \hfill \\ \end{aligned}$$

2 $$\begin{aligned} {\text{Y}} &= \upbeta_{0} + \upbeta_{ 1} {\text{A}} + \upbeta_{ 2} {\text{B}} + - \upbeta_{ 2} {\text{C}} \hfill \\ & \quad + \upbeta_{ 4} {\text{D}} + - \upbeta_{ 5} {\text{E}} + \upbeta_{ 1} \upbeta_{ 2} {\text{AB}} \hfill \\ & \quad + - \upbeta_{ 1} \upbeta_{ 3} {\text{AC}} + - \upbeta_{ 1} \upbeta_{ 4} {\text{AD}} + \upbeta_{ 1} \upbeta_{ 5} {\text{AE}} \hfill \\ & \quad + \upbeta_{ 2} \upbeta_{ 3} {\text{BC}} + \upbeta_{ 2} \upbeta_{ 4} {\text{BD}} + - \upbeta_{ 2} \upbeta_{ 5} {\text{BE}} \hfill \\ & \quad + - \upbeta_{ 3} \upbeta_{ 4} {\text{CD}} + \upbeta_{ 3} \upbeta_{ 5} {\text{CE}} + - \upbeta_{ 4} \upbeta_{ 5} {\text{DE}} \hfill \\ \end{aligned}$$

where Y = response variable, β_0_ = intercept, β_1_, β_2_, β_3_, β_4_, β_5_ = linear coefficients, β_1,2_, β_1,3_, β_1,4_, β_1,5_, β_2,3_, β_2,4_, β_2,5_, β_3,4_, β_3,5_, β_4,5_ = interaction coefficients, β_1_^2^, β_2_^2^, β_3_^2^, β_4_^2^, β_5_^2^ = squared coefficients and A, B, C, D, E, AB, AC, AD, AE, BC, BD, BE, CD, CE, DE = level of independent variables. The determination of statistical significance of model was done by Fisher’s test value, and the multiple coefficient of determination, R squared (R^2^) value has given the proportion of variance explained by the model.

### Cellulase purification

The culture filtrate was subjected to cooling centrifugation (4 °C) at 10,000 rpm to remove the cells debris. Supernatant or crude enzyme extract was subjected to ammonium sulphate precipitation, ion exchange chromatography and gel filtration chromatography as per methodology suggested by Gaur and Tiwari (2015) and to aqueous two-phase system preparation (ATPS) by using Triton X-114 (TX-114) and PEG using methodology suggested by Herculano et al. (2012). Protein concentration and enzyme activity after purification were calculated by Lowry et al. (1951) and Mandels and Weber (1969) respectively.

Partition coefficient of cellulase activity was calculated by using the formula K = Ct/Cb, where Ct is the total enzyme activity of top phase and Cb is the total enzyme activity of bottom phase. The phase volume ratio is defined as, R = V_t_/V_b_, where V_t_ and V_b_ are the volume of top and bottom phase respectively. Yield (Y %) of cellulase enzyme was calculated as Y_Top_ (%) = (Enzyme in top phase/Total amount of enzyme) × 100. Purification factor (PF) of cellulase in the top phase was calculated as PF_Top_ = (Specific activity of cellulase at top phase/Specific activity of crude cellulase), where specific activity represents the ratio of total enzyme activity to total protein concentration in the sample.

### Gel electrophoresis and zymography

SDS-PAGE was done according to Laemmli (1970). Protein bands were analysed by coomassie brilliant blue R-250 staining method. For the detection of cellulase activity using zymography, 0.1% (w/v) CMC was added to the polyacrylamide gel. Subsequently, the gel was incubated in 50 mM acetate buffer, pH 5 at 50 °C for 1 h to allow the enzyme to hydrolyse the substrate (CMC), followed by staining with 0.1% (w/v) congo red solution and de-staining with 1 M sodium chloride. De-staining reaction was stopped by immersing the gel in 0.1% (v/v) glacial acetic acid solution.

### Thermal, pH stability and kinetic parameters of partially purified cellulase

The temperature and pH stability for the partially purified enzyme was determined. The pH stability was determined by measuring the enzyme activity by incubating enzyme in different buffers i.e., acetate (pH 3–6), phosphate (pH 7–8), and glycine-sodium hydroxide (pH 9–10) for up to 24 h. The thermal stability for cellulase activity was determined by incubating the enzyme over a temperature range of 25–65 °C for up to 24 h. At regular intervals of 4 h, samples were withdrawn to determine the relative enzyme activity. The K_m_ and V_max_ were studied using the standard reaction mixture of substrate and enzyme. The substrate CMC was in the concentration range from 0.1 mg/mL to 1 mg/mL. The K_m_ and V_max_ were calculated from the graph according to Lineweaver-Burk (double-reciprocal) plot of 1/v against 1/[S] giving intercepts at 1/V_max_ and − 1/K_m,_ Where S and V are substrate concentration and enzyme velocity respectively.

### Application of cellulase and xylanase separately and in combination for the hydrolysis of rice straw and wheat bran

Rice straw and wheat bran were washed, dried and sieved to 2–5 mm particle size. The agro-residues were suspended in sodium acetate buffer (50 mM, pH 4.5) at 2% (w/v) and supplemented with xylanase enzyme from *Aspergillus oryzae,* cellulase enzyme from *S. commune* NAIMCC-F-03379 (100 IU/g of substrate) and cocktail of cellulase and xylanase enzyme. The reaction mixtures were then incubated at 50 °C. The total reaction volume was 10 mL. Every 2 h, the aliquotes of the reaction mixture were withdrawn, centrifuged at 5000 rpm at 4 °C for 15 min, and the supernatants were used for the quantification of the reducing sugar content by the 3,5-dinitrosalicylic acid method using glucose and xylose as a standard. The reducing sugars (glucose and xylose) in the saccharification mixture were determined for 2–10 h and the time profiles for the saccharification of the agro-residues were plotted.

### Analysis of hydrolysed products by TLC

Rice straw and wheat bran were subjected to enzymatic hydrolysis using commercial cellulase (ONZUKAR), in-house produced cellulase, xylanase and xylanase–cellulase cocktail. The samples were withdraw after 8 h of incubation followed by centrifugation at 5000 rpm at 4 °C for 15 min, and the supernatants were used for ascending TLC. The analysis of reducing sugars by TLC was performed on silica gel plates using 1-butanol/ethanol/water (5:3:2, v/v/v) as mobile phase. The supernatant was loaded in silica gel plates, with glucose as control. Plates were air dried at room temperature and sprayed with aniline diphenylamine reagent. Plates were incubated at 100 °C for 1 h and blue spots were observed.

### Determination of monosaccharide/disaccharide after enzymatic hydrolysis of rice straw by cellulase obtained from *S. commune* NAIMCC-F-03379

Rice straw was washed, dried and sieved to 2–5 mm particle size and subjected to enzymatic hydrolysis using the partially purified cellulase obtained from *S. commune* NAIMCC-F-03379. After enzymatic hydrolysis the supernatant was subjected to HPLC. The supernatant was analyzed through HPLC using an “Agilent Hi-Plex Pb column” (7.7 × 100 mm, 8 μm) and 1290 Infinity LC II system (Agilent Technologies, Santa Clara, CA, USA). Deionised water (100%) was used as the eluent. The flow rate and operating temperature used were 0.5 mL/min and 70 °C, respectively. The chromatogram peaks exhibited by the samples were identified and quantified based on the retention times of monosaccharide/disaccharide standards (Sluiter et al. 2008).

## Results

### Isolation, screening and identification of cellulase producing fungal strain

A total 20 fungal cultures were isolated. The strains were subjected to plate and quantitative assays using CMC as the sole carbon source. A strain designated COC exhibited good capability of hydrolyzing CMC on czapek dox agar. Based on the morphological and molecular identification, the strain was identified as *S. commune* (Additional file 1: Fig. S1). The culture *S. commune* was deposited with accession number NAIMCC-F-03379 at ICAR-National Agriculturally Important Microbial Culture Collection (NAIMCC), Kushmaur, Mau Nath Bhanjan, Uttar Pradesh, India. The sequence for the *S. commune* was submitted to GenBank with accession number MG923341.1

### Selection of medium for cellulase production

**Fig. 1:**
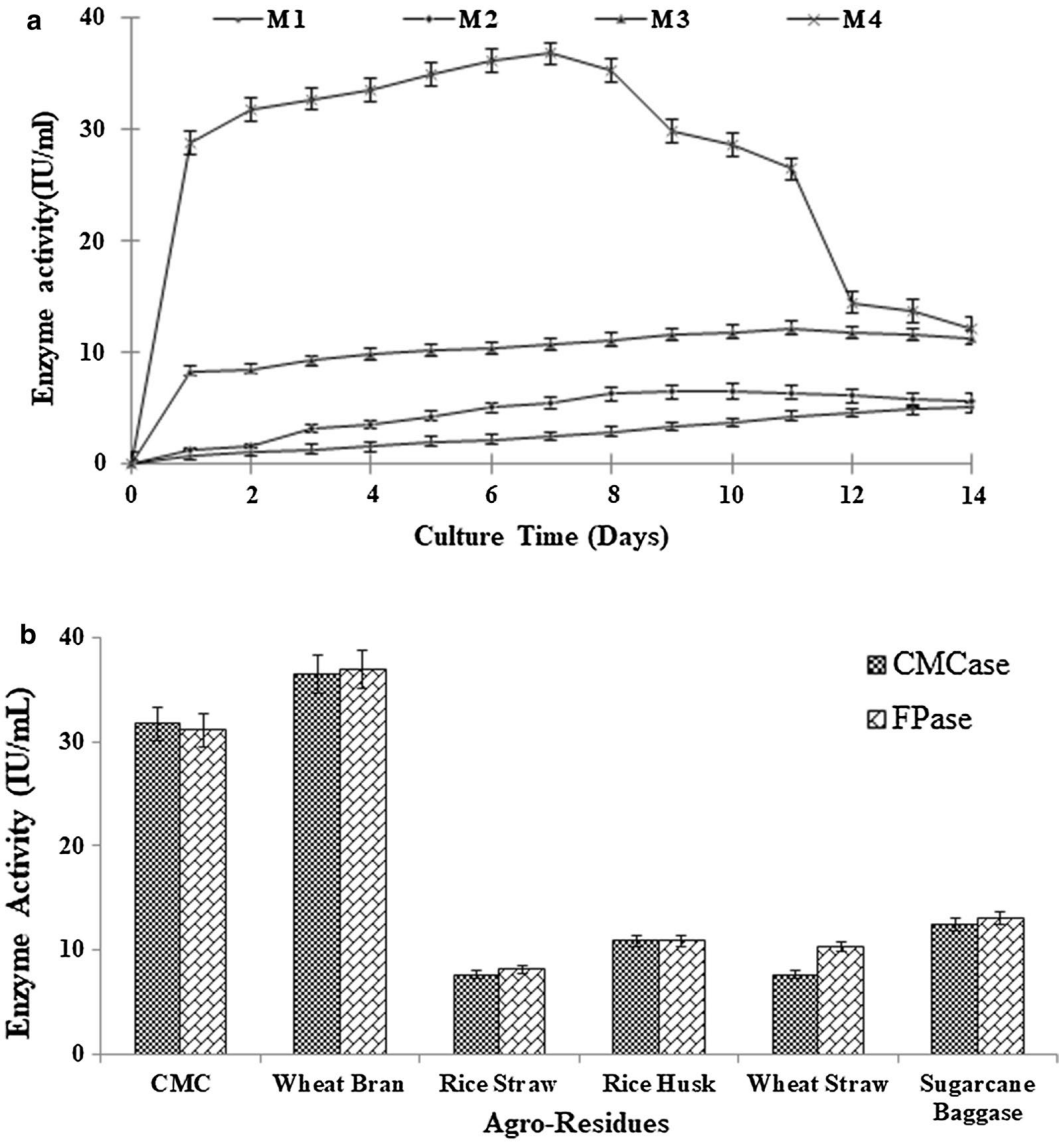
Cellulase production using different media and agro-residues. **a** Cellulase production by *Schizophyllum commune* NAIMCC-F-03379 using different media (M1, M2, M3 and M4). **b** Selection of agro-residues for cellulase production by *Schizophyllum commune* NAIMCC-F-03379 in M4 Media

The selected strain *S. commune* NAIMCC-F-03379 was subjected to submerged fermentation for cellulase production using different medium i.e. M1, M2, M3 and M4 at pH 5 and temperature of 28 °C under static condition. The maximum CMCase activity of 36.8 IU/mL was obtained in M4 medium on 7 days of incubation using 1% CMC as the sole carbon source (Fig. 1a).

### Selection of agro-residue for cellulase production

Five different type of agro-residues RS, RH, WS, WB and SB were substituted as the sole carbon source in MSBS M4 media. *S. commune* NAIMCC-F-03379 exhibited maximum CMCase and FPase activity of 36.46 IU/mL and 37.01 IU/mL respectively on 2nd day of incubation (Fig. 1b) using wheat bran as sole carbon source. The CMCase and FPase activity obtained were comparable to CMC as a substrate but much higher than the other agro-residues. The CMCase activity obtained when RS, RH, WS and SB used as substrates were 7.6 IU/mL, 10.9 IU/mL, 7.6 IU/mL and 12.5 IU/mL, respectively. Similarly the FPase activity of 8.2 IU/mL, 10.9 IU/mL, 10.3 IU/mL and 13.1 IU/mL was obtained using RS, RH, WS, and SB respectively.

### Statistical optimization of nutrient and physical parameters for enhanced cellulase production

In the present work, RSM was employed using CCD to obtain a quadratic model. The independent variables were concentration of wheat bran (A), concentration of magnesium sulphate (B), concentration of calcium chloride (C), temperature (D), and pH (E). The experimental design was generated by design expert software. Table 2 summarizes the central composite experimental plan, as well as predicted and observed CMCase and FPase responses respectively for each experiment.

**Table 2:** Experimental design with coded levels of variables used in CCD-RSM with predicted and experimental CMCase and FPase activity for *Schizophyllum commune* NAIMCC-F-03379

Run	Space type	Coded levels					CMCase		FPase	
		X1	X2	X3	X4	X5	Experimental	Predicted	Experimental	Predicted
1	Factorial	− 1	− 1	1	− 1	− 1	60.57	58.49	65.33	78.75
2	Factorial	− 1	− 1	− 1	1	1	40.11	40.98	58.72	51.99
3	Factorial	0	0	1	0	0	85.78	83.89	69.21	64.99
4	Factorial	− 1	− 1	1	1	1	47.17	45.23	64.09	64.42
5	Factorial	0	0	0	1	1	55.81	54.71	61.80	75.96
6	Factorial	0	− 1	− 1	1	− 1	49.90	47.87	169.98	153.70
7	Factorial	0	− 1	1	− 1	0	72.82	74.69	64.18	61.15
8	Factorial	− 1	1	1	1	− 1	46.73	46.65	75.03	69.20
9	Factorial	1	− 1	0	0	− 1	84.99	84.03	70.27	66.48
10	Factorial	− 1	1	0	− 1	0	59.51	61.44	61.27	75.58
11	Factorial	1	0	− 1	− 1	1	88.60	91.59	77.94	80.41
12	Factorial	− 1	− 1	1	1	− 1	47.78	47.67	55.37	51.92
13	Factorial	0	− 1	− 1	− 1	1	72.03	66.39	65.95	64.13
14	Factorial	0	− 1	1	1	1	55.45	57.70	55.81	56.17
15	Factorial	− 1	− 1	1	− 1	1	54.93	57.98	87.63	89.75
16	Factorial	− 1	1	1	− 1	− 1	60.22	61.00	74.67	67.95
17	Factorial	0	− 1	− 1	− 1	− 1	72.21	73.71	63.04	62.30
18	Factorial	0	1	1	1	− 1	45.49	48.45	73.70	89.82
19	Factorial	0	− 1	1	1	− 1	48.75	47.26	52.46	43.50
20	Factorial	1	1	− 1	1	0	65.33	67.38	57.13	59.86
21	Factorial	0	1	− 1	− 1	− 1	68.15	66.18	62.68	68.06
22	Factorial	− 1	− 1	− 1	− 1	− 1	60.04	60.79	58.54	50.31
23	Factorial	− 1	1	1	1	1	47.87	47.86	56.51	55.14
24	Factorial	0	1	1	− 1	1	96.72	93.40	62.42	58.41
25	Factorial	0	1	− 1	0	1	67.97	70.82	59.86	64.76
26	Factorial	− 1	1	− 1	1	− 1	51.05	49.81	54.75	53.63

The regression equation was obtained after ANOVA provided an estimate of the level of CMCase and FPase activity as a function of the independent variables. The production of cellulase (CMCase and FPase) enzyme incorporating the interactions of different levels of each variable may be predicted best by the following polynomial mathematical model (Eqs. 3, 4),

3 $$\begin{aligned} \left( {\text{For CMCase}} \right){\text{ Day 1}} & = \left( { 78.4247} \right) + \left( {12.519 1\times {\text{A}}} \right) + \left( { - 0. 6 7 1 9 1 3\times {\text{B}}} \right) \hfill \\ & \quad + \left( { 4. 1 7 8 2 9\times {\text{C}}} \right) + \left( { - 10.7812\times {\text{D}}} \right) + \left( { 1. 6 9 2 9 7\times {\text{E}}} \right) + \left( { 1. 10 7 7 4\times {\text{AB}}} \right) \hfill \\ & \quad + \left( {0. 6 2 5 2 8 5\times {\text{AC}}} \right) + \left( { - 7. 2 8 6 1 5\times {\text{AD}}} \right) + \left( { 6.43701\times {\text{AE}}} \right) + \left( { 3.0 6 5 6 1\times {\text{BC}}} \right) \hfill \\ & \quad + \left( { - 0. 8 8 5 1 8 1\times {\text{BD}}} \right) + \left( {0. 9 1 2 1 8 5\times {\text{BE}}} \right) + \left( { - 3. 2 80 4 5\times {\text{CD}}} \right) + \left( { 4. 9 20 5 8\times {\text{CE}}} \right) \hfill \\ & \quad + \left( { - 0. 4 8 1 6 2 6\times {\text{DE}}} \right) + \left( { - 1. 2 7 9 6 7\times {\text{A}}^{ 2} } \right) + \left( { - 2. 80 60 2\times {\text{B}}^{ 2} } \right) + \left( { 1. 2 8 9 7 9\times {\text{C}}^{ 2} } \right) \hfill \\ & \quad + \left( { - 1 7. 1 7 6 5\times {\text{D}}^{ 2} } \right) + \left( { 4. 1 4 1 7 5\times {\text{E}}^{ 2} } \right) \hfill \\ \end{aligned}$$

4 $$\begin{aligned} \left( {\text{For FPase}} \right){\text{ Day 4}} & = \left( { 6 9. 2 4 3 3} \right) + \left( { 1 6. 3 9 3\times {\text{A}}} \right) + \left( { 6. 3 80 3 9\times {\text{B}}} \right) \hfill \\ & \quad + \left( { - 4. 2 4 9 3 6\times {\text{C}}} \right) + \left( { 7. 70 2 9 7\times {\text{D}}} \right) + \left( { - 3.0 1 5 2 4\times {\text{E}}} \right) \hfill \\ & \quad + \left( {14. 5 20 4\times {\text{AB}}} \right) + \left( { - 1 7. 5 8 7 1\times {\text{AC}}} \right) + \left( { 7. 3 8 3 6 8\times {\text{AD}}} \right) \hfill \\ & \quad + \left( {0.0 8 6 4 6 5 3\times {\text{AE}}} \right) + \left( { 3. 1 1 9 1 7\times {\text{BC}}} \right) + \left( { 7.0 2 1 3 7\times {\text{BD}}} \right) \hfill \\ & \quad + \left( { - 6. 6 4 10 4\times {\text{BE}}} \right) + \left( { - 6. 3 3 9 1 6\times {\text{CD}}} \right) + \left( { 2. 3 3 3 8 9\times {\text{CE}}} \right) + \left( {0. 3 7 6 3 4 6\times {\text{DE}}} \right) \hfill \\ \end{aligned}$$

where Y = enzyme production, A = wheat bran, B = magnesium sulphate, C = calcium chloride, D = temperature, and E = pH.

Statistical analysis was employed with Fisher’s f-test and Student’s t-test. Additional file 1: Table S1 shows the summary of the ANOVA for the response surface quadratic model.

The Model F-value for CMCase activity was 11.04, which implies that the model is significant. There is only a 0.72% chance that an F-value this large could occur due to noise. Values of “Prob > F” less than 0.0500 indicate model terms are significant. In this case A, D, AD, and CE, D^2^ are significant model terms. Similarly the Model F-value for FPase activity was 4.75 which implies the model is significant. There is only a 0.86% chance that an F-value this large could occur due to noise. Values of “Prob > F” less than 0.0500 indicate model terms are significant. In this case A, D, AB, AC, BD is significant model terms. Model terms to be significant, it values must be less than 0.1000. (Additional file 1: Table S2). In the present study, coefficient determination (R^2^), adjusted R^2^, predicted R^2^, adequate precision and ‘Lackof Fit’ were taken into consideration. The R^2^ value for CMCase activity was calculated as 0.9779 which indicates that the model could explain 97.79% of the variability in the response (Table 3). The R^2^ value for FPase activity was calculated as 0.8770 which indicates that the model could explain 87.70% of the variability in the response (Table 3). A negative “Predicted R-Squared” implies that the overall mean is a better predictor of the response than the current model “Adequate Precision” measures the signal to noise ratio. A ratio greater than 4 is desirable. The ratio of 11.705 (CMCase) and 11.357 (FPase) indicates an adequate signal. This model can be used to navigate the design space.

**Table 3:** Coefficient for determination (R^2^) for CMCase and FPase activity of *Schizophyllum commune* NAIMCC-F-03379

Parameters	CMCase	FPase
Std. dev.	4.98	12.37
Mean	61.77	68.40
C.V. %	8.07	18.08
PRESS	9385.93	18533.40
R-squared	0.9779	0.8770
Adjusted R-squared	0.8893	0.6925
Predicted R-squared	− 0.6740	− 0.4899
Adequate precision	11.705	11.357

Interaction effect of variables on cellulase yield was studied against any two variables while keeping other independent variables at a constant level. Given response surface plots and contour plots can be utilised for predicting the optimal values for various test series. 3D response surface plots and contour plots showed interaction between temperature and wheat straw, pH and calcium chloride that were most possible combinations obtained from the response evaluation. Contour plots study explains the optimal value of four most effective variables which lies between the following ranges: wheat bran = 0.8–1.0%, pH = 4–5, calcium chloride 0.5–0.7% and temperature 25–30 °C.

**Fig. 2:**
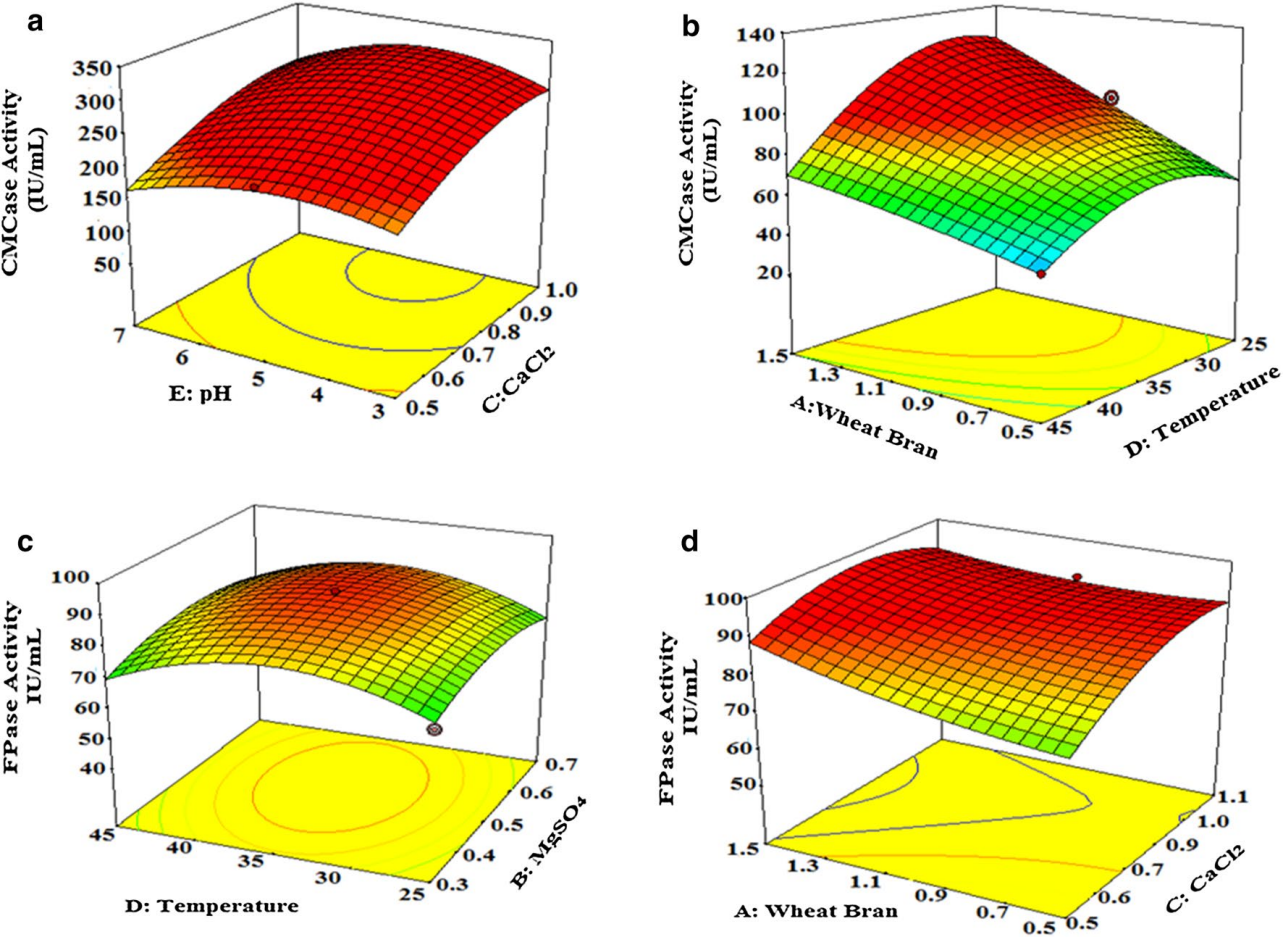
Response surface plot and contour plot for CMCase and FPase production from *Schizophyllum commune* NAIMCC-F-03379 showing interaction between different variable: For CMCase activity **a** pH and calcium chloride, **b** wheat bran and temperature; For FPase activity **c** temperature and magnesium sulphate, **d** wheat bran and calcium chloride

The 3D response surface plot for cellulase activity is presented in the Fig. 2. At concentration of wheat bran (0.8–1.0%) and at temperature range 25 ± 1 °C the enzyme activity was high (> 80 IU/mL) (Fig. 2b). When concentration of wheat bran was decreased below 1% and temperature was kept above 35 °C, activity was decreased (< 50 IU/mL). Upon moving from pH 3 towards pH 5, increase in the enzyme activity was observed. All these five factors have individual importance in cellulase production (Fig. 2). The concentration of most effective interacting components can be changed on the contour plot for the cost-effective production of cellulase from the industrial point of view.

### Validation of the experimental model

The optimum values for the three most effective variables as per the cube plot were determined to be 0.3 g/L magnesium sulphate, 1% (w/v) WB, and 25 °C temperature. The calcium chloride concentration was 0.8–1.0 g/L and the initial pH of the medium was set to 5. The validity of the model was confirmed by using the optimized values for all of the variables in the MSBS medium for production of the cellulase enzyme. The validation experiments suggested that 1% RS, 0.3 g/L MgSO_4_, 1.0 g/L CaCl_2_, 25 °C, and pH of 5 were the optimum conditions for cellulase production. These conditions led to a CMCase and FPase activity of 96.72 IU/mL, and 169.98 IU/mL respectively, which were comparable to the model predicted values of 93.40 IU/mL and 153.70 IU/mL. Hence, the CCD based RSM models were considered to be accurate and reliable for optimizing the cellulase production.

### Cellulase production under shaking conditions

Cellulase production using the un-optimized and optimized parameters under shaking conditions (100 rpm) was conducted in order to assess the effect of shaking on the enzyme production. For the un-optimized parameters, the maximum CMCase and FPase activity of 36.46 IU/mL and 37.01 IU/mL respectively was achieved on 2nd day of incubation. The maximum CMCase and FPase activity 195 ± 3.5 IU/mL and 245 ± 1.12 IU/mL was obtained with the optimized parameters after 1 and 4 days of incubation respectively. Therefore, the optimization resulted in 5.35-fold increase in CMCase and 6.62-fold increase in FPase production under shaking condition.

### Comparative study of different purification strategies of crude cellulase enzyme

The crude enzyme extract was subjected to ammonium sulphate precipitation. Maximum activity was observed in the fraction obtained by addition of 60% ammonium sulphate with protein concentration of  13.9 mg/mL. This fraction had 73 IU/mg of specific activity and with regards to purity it showed 6.1-fold purification (Table 4).

**Table 4:** Comparative analysis of different purification strategies for the cellulase from *Schizophyllum commune* NAIMCC-F-03379

Technique	Purification step	Enzyme activity (IU/mL)	Protein (mg/ml)	Vol. (mL)	Total activity (IU)	Total protein (mg)	Specific activity	Yield (%)	PF
Ammonium sulphate precipitation	Crude	236.4	19.8	500	118,193.9	9882.00	12.0	100	1
	60% (NH_4_)_2_SO_4_	1011.7	13.9	13	13,151.8	180.18	73.0	11.1	6.1
Anion exchange chromatography	Crude	236.4	19.8	5	1181.9	98.80	12.0	100	1
	400 mM Fraction	242.4	5.0	2	484.9	10.08	48.1	41.0	4.0
Gel filtration chromatography	Crude	236.4	19.8	3	709.2	59.40	12.0	100	1
	0 – 10 ml Fraction	103.3	2.3	5	516.6	11.55	44.7	72.8	3.7
ATPS with TX-114	Crude	236.4	19.8	4	945.6	79.20	12.0	100	1
	8% (v/v) TX-114	249.6	2.9	3	748.8	8.64	86.7	79.2	7.2
ATPS with MnSO_4_	Crude	236.4	19.8	2	472.8	39.60	12.0	100	1
	22.5% salt 11.3% PEG 8000	93.9	0.8	4	375.8	3.00	125.3	79.5	10.4

Crude enzyme was subjected to each purification methods separately (Single Step Purification). Purification fold with respect to the crude enzyme. The respective enzyme load in each purification technique was mentioned in methodology

The crude enzyme extract was subjected to ion exchange chromatography. Sample was loaded onto DEAE Sephadex A-50 column pre-equilibrated with sodium acetate buffer (50 mM, pH 5) and allowed to pass through column. The unbound fraction was collected and analyzed for cellulase activity and protein content. There was no cellulase activity in the unbound fractions. The absence of enzyme in the unbound fraction suggested that total cellulase was bound to matrix. The bound enzyme was eluted by sodium acetate buffer having NaCl with increasing concentration at gradients of 0.1 M. 2 mL of each concentration of NaCl was used to evade the bound enzyme. The cellulase activity was detected in the fraction released by the addition of 0.4 M NaCl. Cellulase on column resulted in several peaks with 3 prominent peaks at 8th, 30th and 42nd fraction (Additional file 1: Fig. S2A). The enzyme activity profile was given in Additional file 1: Fig. S2B. The maximum enzyme activity was obtained in the fraction 10–20, with 4-fold purification and 41% yield.

The crude enzyme was subjected to gel filtration chromatography where crude enzyme extract was loaded into a Sepharose G-100 column. Enzyme elution was carried out by using 50 mM sodium acetate buffer (pH 5.0). Each fraction was analysed for cellulase activity and protein content. The elution profile of cellulase purification on Sepharose G-100 had the cellulase elute showing several peaks with three prominent peaks at 9th, 35th and 42nd fraction (Additional file 1: Fig. S2C). The maximum purification was obtained in the 0–10 fraction (Additional file 1: Fig. S2D) of elution with yield of 72.8% and 3.7-fold purification.

The crude xylanase enzyme was subjected to an aqueous two-phase system using the Triton X-114 aqueous phase system. The concentration range of Triton X-114 tested was 2-16% (v/v). Maximum purification was obtained with 8% TX-114 concentration, with 7.2-fold purification and 79.2% yield. With increase in concentration above 16%, no partition was observed.

Another aqueous two-phase system used was PEG of molecular weight 6000 and 8000 in combination with different sulphate salts such as ZnSO_4_, MnSO_4_, FeSO_4_, CuSO_4_, (NH_4_)_2_SO_4_, Na_2_SO_4_, K_2_SO_4_, CaSO_4_ and MgSO_4_ was tested. Among all tested system 11.3% PEG 8000 and 22.5% MnSO_4_ showed maximum purification yield of 10.4-fold and yield of 79.5% as compared to other techniques reported (Table 4).

Therefore, among various purification strategies, the ATPS (PEG/MnSO_4_) system showed high recovery of 79.5% with 10.4-fold increase in activity with reduced number of downstream processing steps and low cost phase forming materials (Table 4). The partition coefficient of enzyme (K) was 0.5 (Table 5). Therefore, it can be considered as a low cost and feasible alternative technique for cellulase purification. The enzyme obtained by the ATPS (PEG 8000/MnSO_4_) was further used for characterization and hydrolysis studies.

**Table 5:** Partition parameters of ATPS using PEG 8000/MnSO_4_ salt system for purification of cellulase from *Schizophyllum commune* NAIMCC-F-03379

Partition parameters	Value
MnSO_4_ concentration (w/w %)	22.50
PEG 8000 concentration (w/w %)	11.25
Crude enzyme extract (w/w %)	100.00
Phase volume ratio (R)	0.30
Partition coefficient of enzyme (K)	0.50

### SDS-PAGE and zymography

**Fig. 3:**
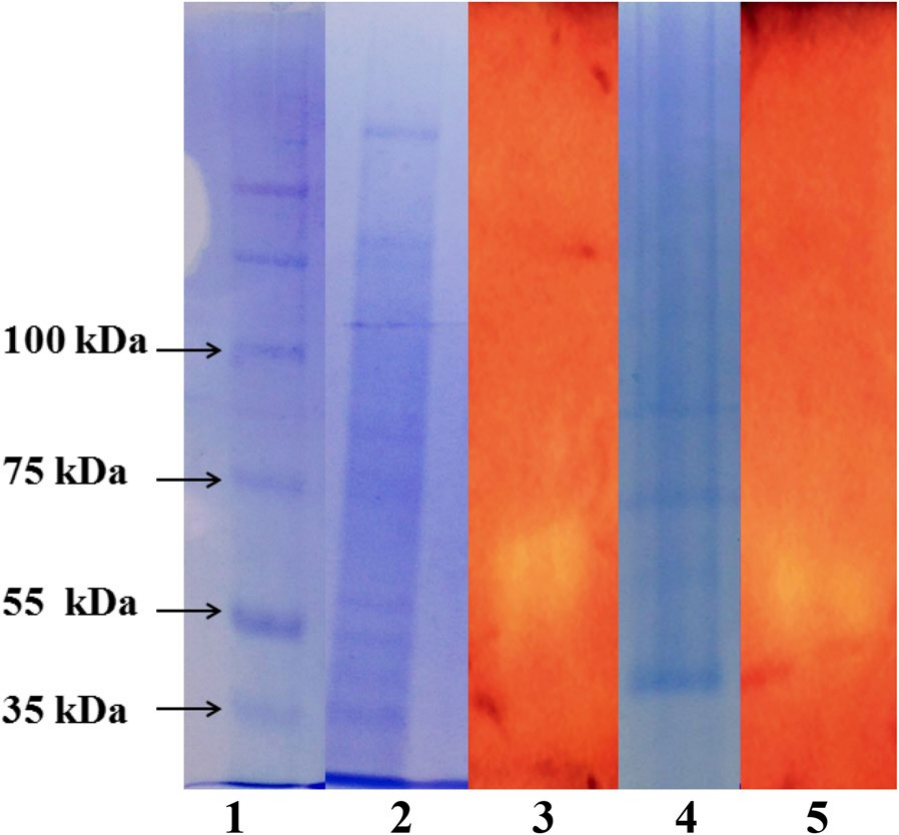
SDS-PAGE analysis and zymography: (1) Protein Ladder, (2) SDS-PAGE of crude cellulase, (3) zymography of crude cellulase, (4) SDS-PAGE of partially purified cellulase enzyme by ATPS with PEG 8000/MnSO_4_ system, (5) Zymography of the partially Purified cellulase enzyme by ATPS with PEG 8000/MnSO_4_ system. CMC was used as substrate in zymography

SDS-PAGE was performed for crude cellulase as well as the enzyme obtained from the best purification techniques i.e. ATPS PEG 8000/MnSO_4_ system. Multiple bands were observed in the crude sample whereas in case of ATPS system only few bands were obtained (Fig. 3). The presence of cellulase on gel was confirmed by performing zymography using 1% CMC as substrate. In the zymograph a clear zone was observed parallel to position approximately ~ 60 kDa (Fig. 3), which resulted due to hydrolysis of cellulose present in substrate CMC. Thus it is evident from zymography of both crude and partially purified enzyme (ATPS PEG 8000/MnSO_4_) that the protein band corresponding to the ~ 60 kDa is cellulase enzyme.

### Thermal, pH stability and kinetic parameters of partially purified cellulase

**Fig. 4:**
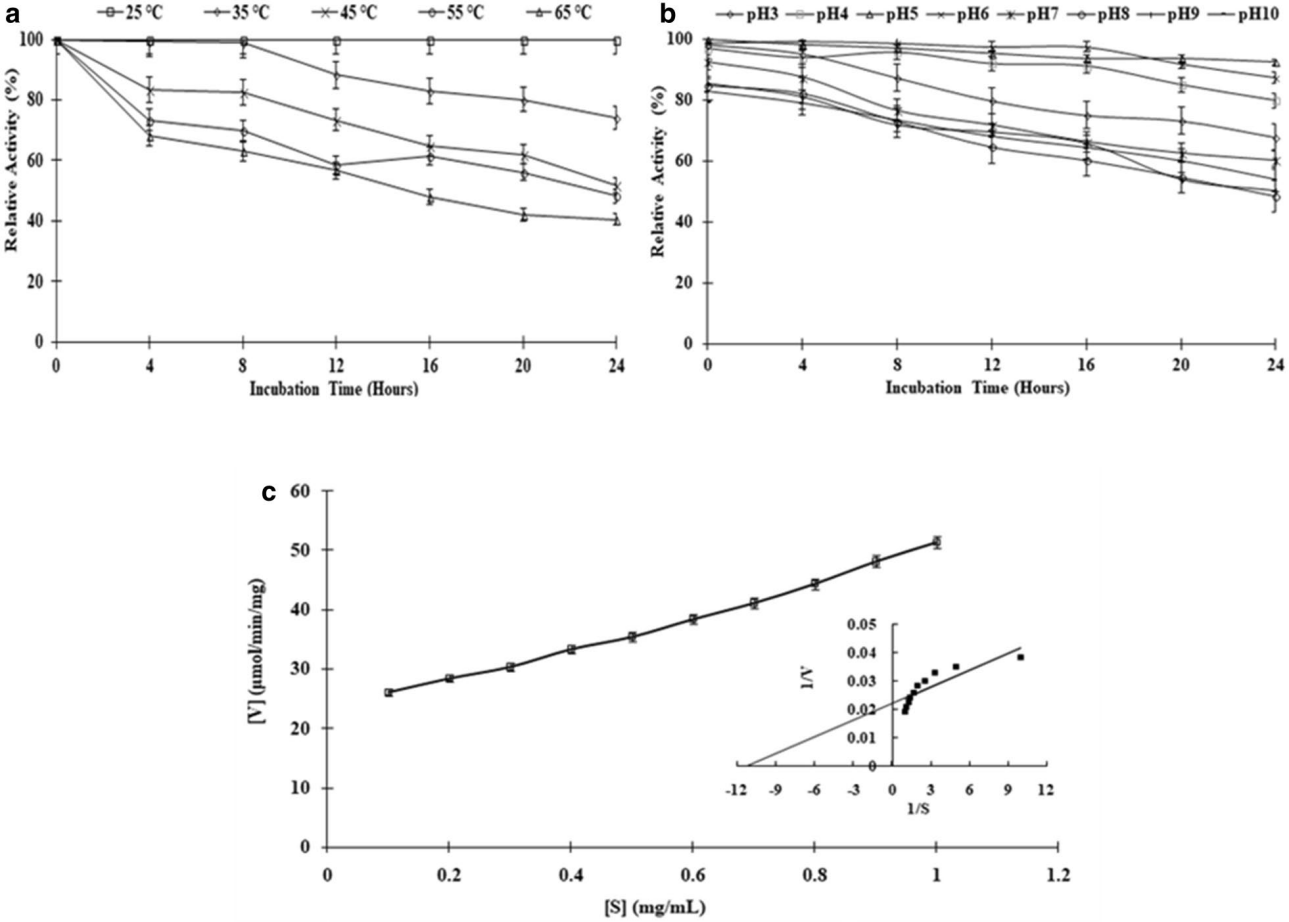
Stability and kinetic analysis of partially purified *Schizophyllum commune* NAIMCC-F-03379: **a** thermal stability of the partially purified cellulase enzyme at different temperature (25–65 °C) for different interval of time. **b** Stability of the partially purified cellulase enzyme produced at different pH (3–10) for different interval of time. **c** Kinetics (Michaelis–Menten and Lineweaver–Burk plots) of cellulase enzyme for CMC as substrate

The pH and thermal stability of the partially purified cellulase enzyme was determined by incubating the enzyme at the requisite pH and temperature, and the CMCase activity was then evaluated. The enzyme showed thermal stability at temperature of 65 °C, as it retained 41% of relative activity (Fig. 4a) and with no loss of activity at 25 °C after 24 h of incubation. The CMCase activity of enzyme decreased gradually with increase in temperature. The partially purified cellulase enzyme of *S. commune* NAIMCC-F-03379 retained more than 53% of its activity in temperature range of 35–55 °C and more than 40% of activity at temperature of 65 °C even after 24 h of incubation (Fig. 4a). The partially purified enzyme showed relative activity of more than 55% after 12 h of incubation in temperature range of 25–65 °C (Fig. 4a).

In the present study the partially purified cellulase enzyme from *S. commune* NAIMCC-F-03379 showed high relative activity over a wide pH range of 3–10, with 53% relative activity at a pH 10 and 51% of relative activity at pH 9 after 24 h of incubation (Fig. 4b). The enzyme was also found to be highly stable at pH-6, where more than 87% relative activity was obtained after 24 h of Incubation (Fig. 4b). The enzyme showed relative activity of more than 55% after 12 h of incubation in pH range of (3–10). V_max_ of partially purified cellulase was found to be 45.45 μmol/min mg. K_m_ of partially purified cellulase was found to be 0.0909 mg/mL, which indicates that enzyme has high-affinity towards CMC due to its low K_m_ value (Fig. 4c).

### Application of cellulase and xylanase in the hydrolysis of rice straw and wheat bran

**Fig. 5:**
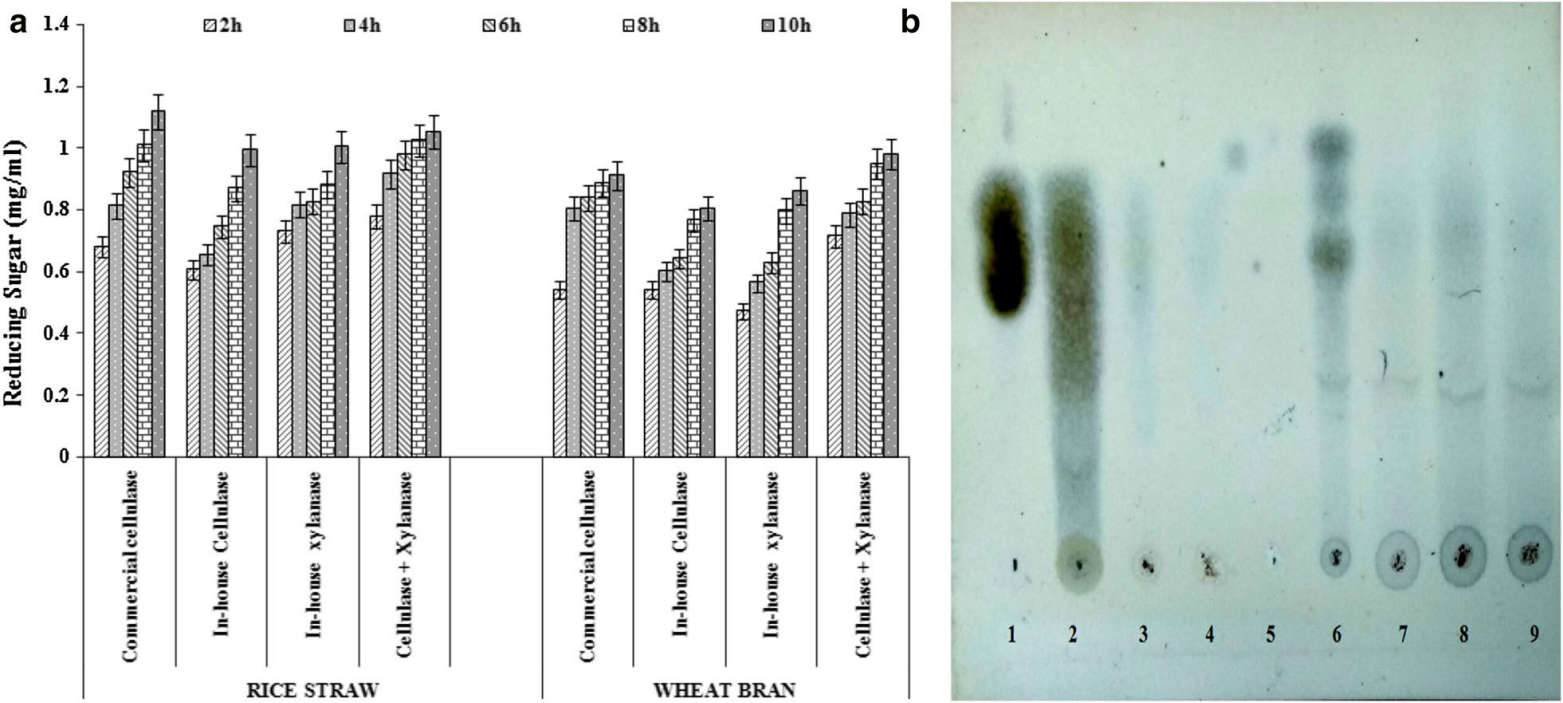
Hydrolysis of rice straw and wheat bran. **a** Hydrolysis of rice straw and wheat bran for the detection of amount of sugar released with time by using commercial cellulase ONZUKAR, cellulase obtained from *Schizophyllum commune* NAIMCC-F-03379, xylanase obtained from *A. oryzae* and combination of cellulase and xylanase. **b** Analysis of hydrolysis product. Glucose as control (1), rice straw by commercial cellulase (2), rice straw by cellulase obtained from *Schizophyllum commune* NAIMCC-F-03379 (3), rice straw by xylanase obtained from *Aspergillus oryzae* (4), rice straw by combination of cellulase and xylanase (5), wheat bran by commercial cellulase (6), wheat bran by cellulase obtained from *Schizophyllum commune* NAIMCC-F-03379 (7), wheat bran by xylanase obtained from *Aspergillus oryzae* (8) and wheat bran by combination of cellulase and xylanase (9) on TLC plates

The cellulase from *S. commune* NAIMCC-F-03379 and xylanase from *A. oryzae* was used separately or in combination for hydrolysis of rice straw and wheat bran. The hydrolysis efficiency of the in-house produced cellulase and xylanase enzymes were comparable to that of commercial cellulase. The maximum sugar concentration of 1.12 mg/mL was observed for hydrolysis of rice straw with commercial cellulase after 10 h of incubation, similarly sugar concentration of 1.05 mg/mL was observed for hydrolysis of rice straw with cocktail of in-house produced cellulase and xylanase. The saccharification yield for wheat bran was less as compared to rice straw however the sugar concentration was more with in-house enzyme cocktail (0.98 mg/mL) as compared to the commercial cellulase (0.91 mg/mL) after 10 h of incubation with respective enzyme (Fig. 5a).

### Analysis of hydrolysed products by TLC

Enzymatic hydrolysis of rice straw and wheat bran was performed using commercial cellulase (ONZUKAR), in-house produced cellulase, xylanase and xylanase-cellulase cocktail. The hydrolysate obtained after 8 h was centrifuged at 5000 rpm and the supernatant is subjected to ascending TLC. Spots were observed on the silica gel plates, which indicated that glucose was generated as a hydrolysis product of wheat bran and rice straw compared with glucose standard (Fig. 5b). The in-house cellulase resulted in glucose generation as comparable to commercial cellulase for both substrates i.e. rice straw and wheat bran. The enzyme cocktail of in-house cellulase and xylanase showed glucose generation as suggested by the TLC as well.

### Determination of monosaccharides/disaccharides after enzymatic hydrolysis of rice straw by cellulase obtained from *S. commune* NAIMCC-F-03379

Sugar analysis in the supernatant obtained from the enzymatic hydrolysis of rice straw by cellulase enzyme obtained from *S. commune* NAIMCC-F-03379 was performed using HPLC. Glucose was the major monomeric sugar constituent present in the hydrolysis sample of rice straw with a retention time of 5.672. The area of glucose peak in sample was 76,278.8 (mAU) and the corresponding glucose concentration obtained after hydrolysis of rice straw with cellulase enzyme was of 1.162 mg/mL (Additional file 1: Fig. S3). The in-house produced cellulase resulted in cellulose hydrolysis  leading to high concentration of glucose from rice straw. The hydrolysis sample also showed peak for other reducing sugars such as di-saccharide cellobiose which results due to action of CBH or exo-glucosidase constituent of cellulase enzyme complex and small concentration of xylose. The presence of xylose also represented that the enzyme produced by the *S. commune* NAIMCC-F-03379 also have some hemicellulolytic enzymes as well.

## Discussion

In this study, cellulolytic fungus COC was isolated from decomposed leaf-sample collected from forests of Assam and identified as *S. commune* NAIMCC-F-03379 based on morphological and molecular identification. Different carbon sources (agro-residues) were screened for their influence on strain *S. commune* NAIMCC-F-03379 and the results revealed that CMC and wheat bran favoured maximum cellulase activity. Gomathi et al. (2012) also reported wheat bran can be used as substrate for cellulase production.

Response surface methodology is a statistical practice that offers mathematical prediction and evaluations for cost-effective experimental designs and offers statistical predictions and evaluations (Singh and Kaur 2012a, b; Jose and Jebakumar 2014; Premalatha et al. 2015). In the present study, RSM was applied with CCD to optimize media components such as wheat bran, MgSO_4_ and CaCl_2_ and physical parameters pH and temperature to improve the cellulase production *S. commune* NAIMCC-F-03379. The optimization experiments, resulted in 5.35-fold increase in CMCase and 6.62-fold increase in FPase production. These results indicated that the model developed for maximizing cellulase production by *S. commune* NAIMCC-F-03379 was reliable and accurate. The developed model was found to be very effective in optimizing the selected medium components evident from *R*^2^ value 0.9779 and 0.8770 for CMCase and FPase respectively. The closer *R*^2^ is to 1, the stronger is the model to predict the response (Chen et al. 2013). The observed *R*^2^ value was comparable with the earlier reports (Muthuvelayudham and Viruthagiri 2010; Wang et al. 2011; Rajeswari et al. 2014). The effects of media components and β-glucosidase (BGL) production have also been studied in *S. commune* KUC9397 by Lee et al. (2014) which resulted in 7.2-fold increase in BGL activity. The requirement for fermentation for *S. commune* NAIMCC-F-03379 for enhanced cellulase activity is comparable to other cellulolytic microorganisms (Pachauri et al. 2016; Premalatha et al. 2015). A valid optimization of microbial enzyme production is possible with the implication of the 3D plots which allows direct visualization of individual and interactive influence of variables (Jabasingh and Nachiyar 2010; Wang et al. 2011; Singh et al. 2014b). The validation of the predicted model was performed and under optimum condition CMCase and FPase activity of 96.72 IU/mL, and 169.98 IU/mL was observed which were comparable to the model predicted value of 93.40 IU/mL and 153.70 IU/mL. The results of the validation studies concur with earlier studies demonstrating the significant roles of organic and inorganic nutrients (Li et al. 2008; Singh et al. 2014b; Premalatha et al. 2015). The effect of shaking on enzyme activity was also studied using the optimum media condition. Under shaking condition the enzyme activity enhanced, with CMCase activity  being comparable to the previous reports where enzyme from *Streptomyces *T3-1 and *Sporothrix carnis* showed CMCase activity of 40.3 IU/mL, 148 IU/mL and 285.7 IU/mL by Jang and Chen (2003), Jang and Chang (2005) and Olajuyigbe and Ogunyewo (2016) respectively. The CMCase and FPase activity of *S. commune* NAIMCC-F-03379 was higher as compared to *S. commune* BCC23363 and *S. commune* mutant G-135 (Sornlake et al. 2017). The CMCase and FPase activity obtained with *S. commune* NAIMCC-F-03379 after optimization  were much higher as compared to the CMcase activity and FPase activity of 17.9 U/mL at 30 °C and 0.34 U/mL at 50 °C respectively by after optimization of concentration of different media components (Desrochers et al. 1981). Kim and Keum (2010) reported FPase activity of 11 FPU which is much lower as compared to the present results.

A single step purification strategy was selected by comparative analysis of efficiency of different purification techniques. The crude enzyme extract was subjected to different purification strategies such as gel filtration chromatography, ion exchange chromatography and aqueous two-phase system. The highest purification efficiency was obtained with ATPS system. The result obtained by the ATPS with Triton X-114 is comparable to purification yield obtained for xylanase enzyme in similar studies performed by Sutay Kocabaş et al. (2015) at 7% Triton X-114 concentration maximum yield of 79% and purification fold of 2.7 was obtained. The ATPS system with 11.3% PEG 8000 and 22.5% MnSO_4_ showed maximum purification yield of 10.4-fold and yield of 79.5% as compared to other techniques reported (Table 4). Among various purification strategies ATPS is an attractive, easy and efficient technique for high recovery of enzyme at low cost. As the purification of enzymes from the crude extract could account up to 70% of the product cost and the cost of hydrolysing enzymes in saccharification of agro-residues is limiting factor in cost effective bioethanol generation.

The crude enzyme and the enzyme obtained after ATPS (PEG 8000/MnSO_4_) purified enzyme was subjected to SDS-PAGE and zymography and based on their results it was evident that protein band corresponding to the ~ 60 kDa was cellulase enzyme.

*Schizophyllum commune* NAIMCC-F-03379 was able to produce comparatively high titre of cellulase enzymes as compared to previous reports (Lee et al. 2014) with characteristics of highly thermal and pH stable, low K_m_ (0.0909 mg/mL) and high V_max_ (45.45 μmol/min mg) value. The partially purified enzyme showed stability at a wide range of temperature (25–65 °C) and pH (3–10) with 41% (65 °C) and 51% (pH 9) of relative activity respectively after 24 h of incubation. Also, the partially purified enzyme showed relative activity of more than 55% after 12 h of incubation in wide temperature (25–65 °C) and pH (3–10) range. This results are comparable to Kim et al. (2005), Samiullah et al. (2009) and Gaur and Tiwari (2015), where they performed studies on pH stability of cellulase obtained from *Bacillus* sp. and found that cellulase are generally stable over a wide range of pH from 4 to 10. The cellulase enzyme from *S. commune* NAIMCC-F-03379 showed high stability over wide range of temperature (25–55 °C) and both acidic and alkali pH range of 3-10, this property can be further exploited for industrial application in bioethanol generation where highly thermo-acid/alkali tolerant cellulase enzyme will be required for simultaneous saccharification of biomass and ethanol generation.

The in-house produced cellulase form *S. commune* NAIMCC-F-03379 and xylanase from *Aspergillus oryzae* used separately and as cocktail to compare its efficiency in agro-residue (rice straw and wheat bran) hydrolysis. Similarly, Kim and Keum (2010) attempted saccharification of hardwood and cellulose by *S. commune*. The cellulase from *S. commune* showed the low saccharification of hardwood but high rate of saccharification on cellulose which was much higher than achieved with a commercial cellulase preparation (Celluclast 1.5 L, 30 FPU/g, glucan). In the present work the cellulase from *S. commune* NAIMCC-F-03379 showed  hydrolysis of different agro residues as observed by TLC and HPLC. The hydrolysate obtained from agro-residues hydrolysis was subjected to TLC and spots were found in the range of glucose standard. The hydrolysate obtained from rice straw hydrolysis was subjected to HPLC that clearly showed the enzyme was capable of efficiently breaking down the cellulose to its monomeric constituents i.e. glucose. The results also suggested that the in-house produced cellulase enzyme showed hydrolytic efficiency comparable to commercial cellulase. Therefore, the present work clearly demonstrates that the isolated strain can be potentially used on industrial scale for cellulase enzyme production using the optimized media and the downstream process suggested in this paper can be used for purification of enzyme which can be subsequently used in agro residues saccharification for bioethanol production.

## Additional file

**Additional file 1: Fig. S1.** Phylogenetic analysis of newly isolated *Schizophyllum commune* NAIMCC-F-03379 using neighbor-joining method. **Fig. S2.** Cellulase purification by Chromatography: DEAE-Sephadex A-50 anion exchange chromatography for cellulase purification (A) Chromatogram (B) CMCase activities for different fractions; Sepharose G-100, gel filteration chromatography for cellulase purification (C) Chromatogram and (D) CMCase activities, for different fraction. **Fig. S3.** Representative chromatogram for overlay of 6 Injections of rice straw (RS) hydrolysed by in-house produced cellulase enzyme. **Table S1.** Analysis of variance for the response surface quadratic model for CMCase and FPase production by *Schizophyllum commune* NAIMCC-F-03379. **Table S2.** Estimated regression coefficient for CMCase and FPase production by *Schizophyllum commune* NAIMCC-F-03379.

## Abbreviations

RSM: response surface methodology
ATPS: aqueous two-phase system
CMC: carboxy-methyl cellulose
TLC: thin layer chromatography
HPLC: high performance liquid chromatography
PDA: potato dextrose agar
RS: rice straw
RH: rice husk
WS: wheat straw
WB: wheat bran
SB: sugarcane bagasse
CCD: central composite design
ANOVA: analysis of variance
