# Supplementary material for: Production, purification and characterization of an acid/alkali and thermo tolerant cellulase from Schizophyllum commune NAIMCC-F-03379 and its application in hydrolysis of lignocellulosic wastes
Source: AMB Express. 2018 Oct 17;8:173. doi: 10.1186/s13568-018-0696-y (PMC6192944; doi:10.1186/s13568-018-0696-y)
Supplement: Supplementary file 1 — Additional file 1: Fig. S1. Phylogenetic analysis of newly isolated Schizophyllum commune NAIMCC-F-03379 using neighbor-joining method. Fig. S2. Cellulase purification by Chromatography: DEAE-Sephadex A-50 anion exchange chromatography for cellulase purification (A) Chromatogram (B) CMCase activities for different fractions; Sepharose G-100, gel filteration chromatography for cellulase purification (C) Chromatogram and (D) CMCase activities, for different fraction. Fig. S3. Representative chromatogram for overlay of 6 Injections of rice straw (RS) hydrolysed by in-house produced cellulase enzyme. Table S1. Analysis of variance for the response surface quadratic model for CMCase and FPase production by Schizophyllum commune NAIMCC-F-03379. Table S2. Estimated regression coefficient for CMCase and FPase production by Schizophyllum commune NAIMCC-F-03379. [file 13568_2018_696_MOESM1_ESM.doc]

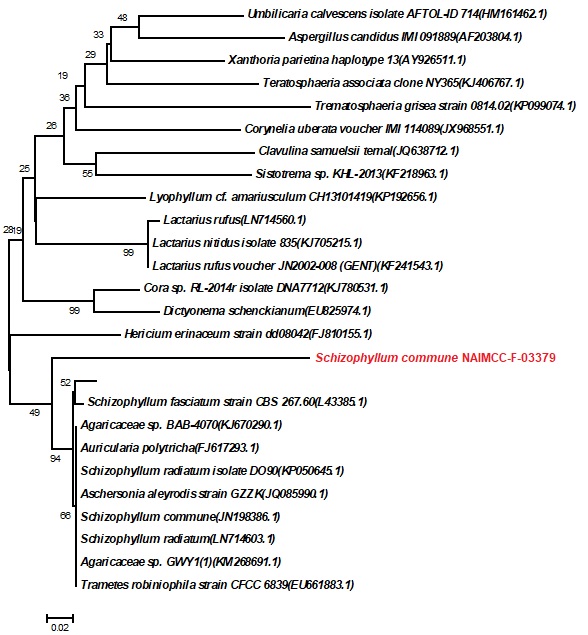


**Fig. S1** Phylogenetic analysis of newly isolated *Schizophyllum commune* NAIMCC-F-03379 using neighbor-joining method


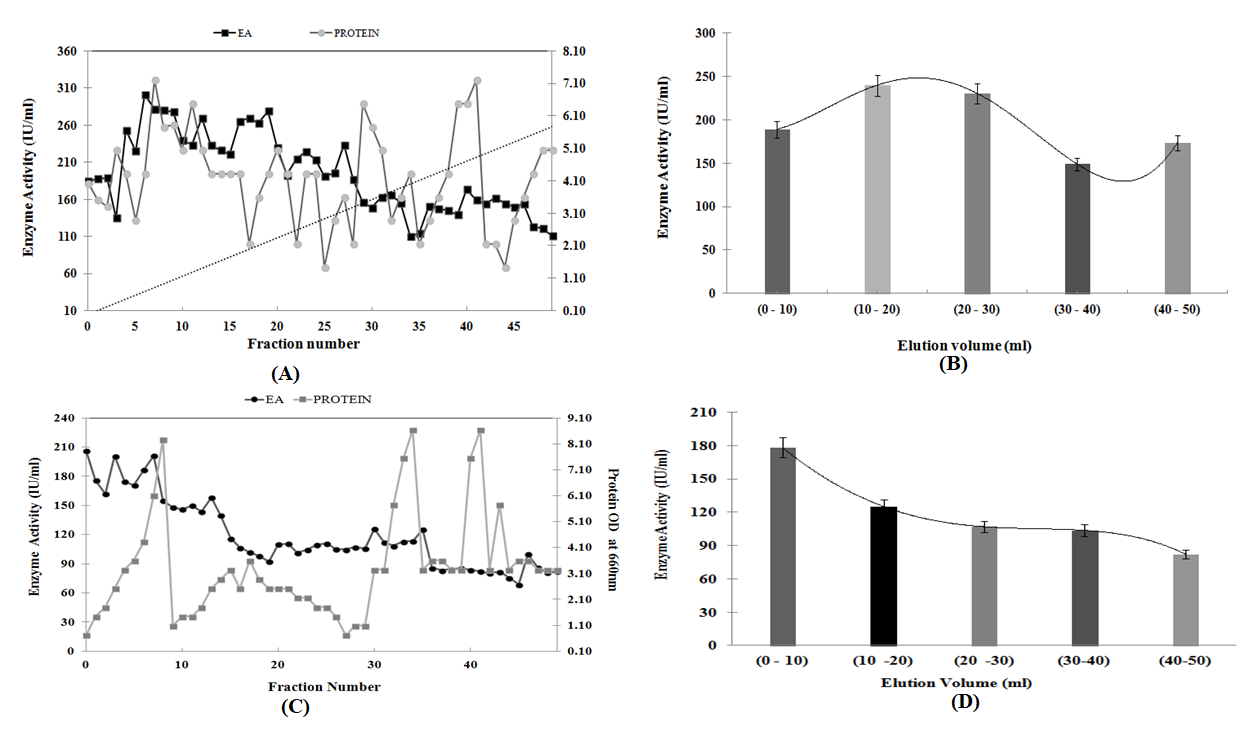


**Fig. S2** Cellulase purification by Chromatography: DEAE-Sephadex A-50 anion exchange chromatography for cellulase purification **(A)** Chromatogram **(B)** CMCase activities for different fractions; Sepharose G-100, Gel filteration chromatogrpahy for cellulase purification **(C)** Chromatogram and **(D)** CMCase activities, for different fraction


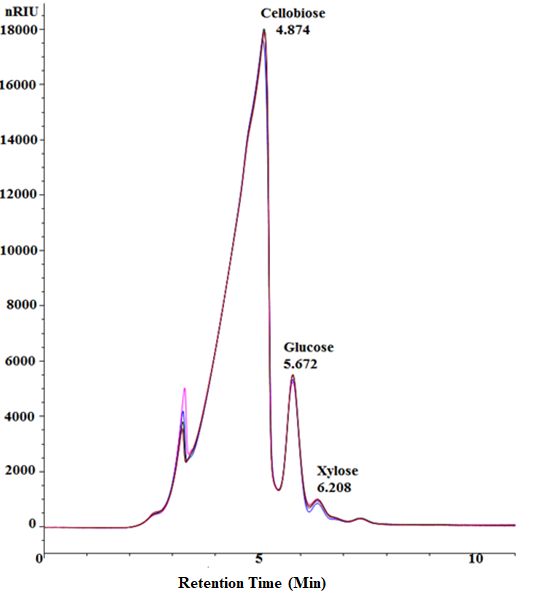


**Fig. S3** Representative chromatogram for overlay of 6 Injections of rice straw (RS) hydrolysed by in-house produced cellulase enzyme

**Table S1 Analysis of variance for the response surface quadratic model for CMCase and FPase production by *Schizophyllum commune* NAIMCC-F-03379**

| **Analysis of variance for the response surface quadratic model for CMCase** | | | | | | |
| --- | --- | --- | --- | --- | --- | --- |
| **Source** | **Sum of**  **Squares** | **df** | **Mean**  **Square** | **F**  **Value** | **p-value**  **Prob > F** |  |
| Model | 5482.76 | 20 | 274.14 | 11.04 | 0.0072 | Significant |
| A-wheat bran | 540.01 | 1 | 540.01 | 21.75 | 0.0055 |  |
| B-Magnesium sulphate | 5.77 | 1 | 5.77 | 0.23 | 0.6500 |  |
| C-Calcium chloride | 158.67 | 1 | 158.67 | 6.39 | 0.0527 |  |
| D-Temperature | 1646.82 | 1 | 1646.82 | 66.32 | 0.0005 |  |
| E-pH | 28.05 | 1 | 28.05 | 1.13 | 0.3365 |  |
| AB | 4.56 | 1 | 4.56 | 0.18 | 0.6861 |  |
| AC | 1.60 | 1 | 1.60 | 0.065 | 0.8096 |  |
| AD | 198.53 | 1 | 198.53 | 7.99 | 0.0368 |  |
| AE | 136.81 | 1 | 136.81 | 5.51 | 0.0658 |  |
| BC | 117.90 | 1 | 117.90 | 4.75 | 0.0812 |  |
| BD | 11.03 | 1 | 11.03 | 0.44 | 0.5347 |  |
| BE | 10.37 | 1 | 10.37 | 0.42 | 0.5466 |  |
| CD | 118.80 | 1 | 118.80 | 4.78 | 0.0804 |  |
| CE | 294.82 | 1 | 294.82 | 11.87 | 0.0183 |  |
| DE | 2.88 | 1 | 2.88 | 0.12 | 0.7472 |  |
| A^2^ | 4.48 | 1 | 4.48 | 0.18 | 0.6886 |  |
| B^2^ | 9.75 | 1 | 9.75 | 0.39 | 0.5584 |  |
| C^2^ | 2.48 | 1 | 2.48 | 0.100 | 0.7649 |  |
| D^2^ | 378.00 | 1 | 378.00 | 15.22 | 0.0114 |  |
| E^2^ | 30.95 | 1 | 30.95 | 1.25 | 0.3150 |  |
| Residual | 124.16 | 5 | 24.83 |  |  |  |
| Core Total | 5606.91 | 25 |  |  |  |  |
| **Analysis of variance for the response surface quadratic model for FPase** | | | | | | |
| **Source** | **Sum of**  **Squares** | **df** | **Mean**  **Square** | **F**  **Value** | **p-value**  **Prob > F** |  |
| Model | 10909.15 | 15 | 727.28 | 4.75 | 0.0086 | Significant |
| A-Wheat bran | 1526.31 | 1 | 1526.31 | 9.98 | 0.0102 |  |
| B-Magnesium sulphate | 597.62 | 1 | 597.62 | 3.91 | 0.0763 |  |
| C-Calcium chloride | 211.87 | 1 | 211.87 | 1.38 | 0.2665 |  |
| D-Temperature | 889.90 | 1 | 889.90 | 5.82 | 0.0366 |  |
| E-pH | 112.64 | 1 | 112.64 | 0.74 | 0.4110 |  |
| AB | 1212.55 | 1 | 1212.55 | 7.93 | 0.0183 |  |
| AC | 2143.56 | 1 | 2143.56 | 14.01 | 0.0038 |  |
| AD | 264.57 | 1 | 264.57 | 1.73 | 0.2179 |  |
| AE | 0.046 | 1 | 0.046 | 3.006E-004 | 0.9865 |  |
| BC | 152.08 | 1 | 152.08 | 0.99 | 0.3423 |  |
| BD | 877.95 | 1 | 877.95 | 5.74 | 0.0376 |  |
| BE | 728.26 | 1 | 728.26 | 4.76 | 0.0541 |  |
| CD | 598.68 | 1 | 598.68 | 3.91 | 0.0761 |  |
| CE | 81.68 | 1 | 81.68 | 0.53 | 0.4818 |  |
| DE | 2.04 | 1 | 2.04 | 0.013 | 0.9103 |  |
| Residual | 1529.99 | 10 | 153.00 |  |  |  |
| Core Total | 12439.14 | 25 |  |  |  |  |

**Table S2 Estimated regression coefficient for CMCase and FPase production by *Schizophyllum commune* NAIMCC-F-03379**

| **Estimated regression coefficient for CMCase** | | | | | |
| --- | --- | --- | --- | --- | --- |
| **Coefficient factor** | **Coefficient**  **Estimate** | **SE coef**  **Error** | **t-value**  **Low** | **High** | **P – value** |
| Intercept | 78.42 | 5.43 | 64.47 | 92.38 |  |
| A-Wheat bran | 12.52 | 2.68 | 5.62 | 19.42 | 0.0072 |
| B-Magnesium sulphate | -0.67 | 1.39 | -4.25 | 2.91 | 0.0055 |
| C-Calcium chloride | 4.18 | 1.65 | -0.071 | 8.43 | 0.6500 |
| D-Temperature | -10.78 | 1.32 | -14.18 | -7.38 | 0.0527 |
| E-pH | 1.69 | 1.59 | -2.40 | 5.79 | 0.0005 |
| AB | 1.11 | 2.58 | -5.54 | 7.75 | 0.3365 |
| AC | 0.63 | 2.46 | -5.70 | 6.95 | 0.6861 |
| AD | -7.29 | 2.58 | -13.91 | -0.66 | 0.8096 |
| AE | 6.44 | 2.74 | -0.61 | 13.49 | 0.0368 |
| BC | 3.07 | 1.41 | -0.55 | 6.68 | 0.0658 |
| BD | -0.89 | 1.33 | -4.30 | 2.53 | 0.0812 |
| BE | 0.91 | 1.41 | -2.72 | 4.54 | 0.5347 |
| CD | -3.28 | 1.50 | -7.14 | 0.57 | 0.5466 |
| CE | 4.92 | 1.43 | 1.25 | 8.59 | 0.0804 |
| DE | -0.48 | 1.41 | -4.12 | 3.15 | 0.0183 |
| A^2^ | -1.28 | 3.01 | -9.02 | 6.46 | 0.7472 |
| B^2^ | -2.81 | 4.48 | -14.32 | 8.71 | 0.6886 |
| C^2^ | 1.29 | 4.08 | -9.21 | 11.79 | 0.5584 |
| D^2^ | -17.18 | 4.40 | -28.49 | -5.86 | 0.7649 |
| E^2^ | 4.14 | 3.71 | -5.40 | 13.68 | 0.0114 |
| **Estimated regression coefficient for FPase** | | | | | |
| **Coefficient Factor** | **Coefficient**  **Estimate** | **Standard**  **Error** | **t-value**  **Low** | **High** | **P-value** |
| Intercept | 69.24 | 2.91 | 62.75 | 75.73 | 0.0086 |
| A-wheat bran | 16.39 | 5.19 | 4.83 | 27.96 | 0.0102 |
| B-Magnesium sulphate | 6.38 | 3.23 | -0.81 | 13.57 | 0.0763 |
| C-Calcium chloride | -4.25 | 3.61 | -12.30 | 3.80 | 0.2665 |
| D-Temperature | 7.70 | 3.19 | 0.59 | 14.82 | 0.0366 |
| E-pH | -3.02 | 3.51 | -10.85 | 4.81 | 0.4110 |
| AB | 14.52 | 5.16 | 3.03 | 26.01 | 0.0183 |
| AC | -17.59 | 4.70 | -28.06 | -7.12 | 0.0038 |
| AD | 7.38 | 5.61 | -5.13 | 19.89 | 0.2179 |
| AE | 0.086 | 4.99 | -11.03 | 11.20 | 0.9865 |
| BC | 3.12 | 3.13 | -3.85 | 10.09 | 0.3423 |
| BD | 7.02 | 2.93 | 0.49 | 13.55 | 0.0376 |
| BE | -6.64 | 3.04 | -13.42 | 0.14 | 0.0541 |
| CD | -6.34 | 3.20 | -13.48 | 0.80 | 0.0761 |
| CE | 2.33 | 3.19 | -4.78 | 9.45 | 0.4818 |
| DE | 0.38 | 3.26 | -6.88 | 7.64 | 0.9103 |
